# A Novel qPCR Assay for the Detection of African Animal Trypanosomosis in Trypanotolerant and Trypanosusceptible Cattle Breeds

**DOI:** 10.1371/journal.pntd.0002345

**Published:** 2013-08-15

**Authors:** Katja Silbermayr, Fuyong Li, Albert Soudré, Simone Müller, Johann Sölkner

**Affiliations:** 1 Institute of Parasitology, Vetmeduni Vienna, Vienna, Austria; 2 Department of Agricultural, Food and Nutritional Science, University of Alberta, Edmonton, Alberta, Canada; 3 Division of Lifestock Sciences, University of Natural Resources and Applied Life Sciences, Vienna, Austria; 4 Institute of Animal Breeding and Genetics, Vetmeduni Vienna, Vienna, Austria; 5 Division of Lifestock Sciences, University of Natural Resources and Applied Life Sciences, Vienna, Austria; IRD/CIRDES, Burkina Faso

## Abstract

This study was conducted to (i) determine the prevalence of African Animal Trypanosomosis (AAT) in tsetse challenged areas, (ii) compare conventional with qPCR detection systems and (iii) evaluate the host genetic background and biology as risk factors. AAT prevalence studies are often confronted with low levels of parasitaemia. Hence, we designed a novel qPCR assay using primers and species specific probes amplifying the Internal Transcribed Spacer 1 (ITS1) gene. Thereby all three AAT species could be detected simultaneously. 368 individuals from three cattle types (Baoulé, Zebu and hybrids) originating from 72 farms in Burkina Faso were analysed. Farmers were interviewed and morphometric measurements of the cattle taken. A chi-squared test and a logistic regression model were calculated to detect associations with infection. In our study, the overall rate of prevalence detected with the novel qPCR assay was 11.14%. Compared to conventional PCR we identified a concordance of 91.30%. We tested 41 animals positive for trypanosome DNA, five animals showed multiple infections. Zebus were twice as often infected (21.74%) compared to Baoulé (9.70%) and hybrids (9.57%). *Trypanosoma vivax* is the dominant species (9.24%), as compared to *T. congolense* (2.44%) and *T. brucei* (0.82%). The chi-squared tests linking the infection events to the breeds (Zebu vs. Baoulé and Zebu vs. hybrids) were on the border of significance. No significant association with other tested parameters could be detected. We introduce a novel qPCR technique for the fast, sensitive and simultaneous detection of the three AAT species. Our results suggest that associations with breed and infection exist since Zebu cattle are more likely to be infected compared to Baoulé and hybrids. Indigenous taurine cattle breeds, like the Baoulé, therefore provide a unique and valuable genetic resource.

## Author Summary

African Animal Trypanosomosis (AAT) is a neglected tropical disease heavily impacting on the poor. Sensitive diagnostic tools are needed since actual parasitaemia levels can be very low, particularly in chronically infected or trypanotolerant animals. Hence, we present a novel real-time PCR (qPCR) assay for the simultaneous detection of the three AAT species (*T. congolense*, *T. brucei* and *T. vivax*). Thereby infected animals can be accurately detected in one step. 368 individuals from three cattle types (Baoulé, Zebu and hybrids) originating from 72 farms in Burkina Faso were analysed. Farmers were interviewed and morphometric measurements of the cattle taken to detect potential risk factors of infection. In our study, the overall rate of prevalence detected with the novel qPCR assay was 11.14% (41/368) compared to 10.87% (40/368) with conventional PCR. Zebus are most often infected (21.74%) compared to Baoulé (9.70%) and hybrid (9.57%) cattle. Except for breed, no significant correlation with other tested parameters could be detected. Baoulé show significantly less infections and therefore provide a unique and valuable genetic resource. In summary, with this novel qPCR technique the three AAT species can be simultaneously detected in a fast and sensitive manner.

## Introduction

Trypanosomiasis affects both humans (sleeping sickness) and animals (nagana) and occurs in 37 sub-Saharan countries. Approximately 60 million people and about 50 million cattle are currently living in risk of infection [1]. The International Livestock Research Institute (ILRI) has listed trypanosomosis among the top ten global cattle diseases impacting on the poor [2]. In tsetse challenged areas of Burkina Faso the African animal trypanosomosis (AAT) is ranked first among nine most important cattle diseases [3]. However, over thousands of years, and presumably under high tsetse challenge, some West African *Bos taurus* cattle breeds have developed a tolerance to trypanosomosis in the course of evolution [4]. One trypanotolerant breed, and thus represents a valuable genetic resource, is the Baoulé cattle. The trypanotolerance character enables them to control the development of parasites and to limit the associated pathological effects and level of parasitaemia [5], [6]. In contrast, zebu (*Bos indicus*) cattle types are more susceptible to trypanosome infections and can only be maintained in tsetse challenged areas through the use of costly trypanocidal drugs.

Three different parasite species, *T. congolense* (subgenus *Nannomonas*), *T. brucei* (subgenus *Trypanozoon*) and *T. vivax* (subgenus *Duttonella*) are causative agents of AAT in cattle [7]. In most areas several trypanosome species can be found in sympatry resulting in single or multiple species infections. African trypanosomes are of great concern for public and animal health, particularly in regions where most of the pathogenic trypanosome species are present. Therefore, the discrimination of the trypanosome species, subspecies or strain can be necessary for medical, sanitary, taxonomic or epidemiological studies [8].

AAT prevalence has been previously assessed microscopically with blood smears [9] or by buffy coat examination [10], [11]. Both methods lack sensitivity and are laborious. ELISA techniques can be applied on large sample sets with accurate precision results but it does not differentiate between present and past infections. Moreover, trypanosome species cannot be discriminated with these serological-based detection methods [12]. Following the increasing demand for accurate, fast, sensitive and efficient detection tools, molecular methods have been steadily improved in recent years.

To identify the most appropriate detection system, prevalence studies comparing several techniques were performed. In Sideradougou, Burkina Faso a survey of the agro-pastoral zone showed that the parasitological prevalence in cattle detected with the buffy-coat method was 5.3% compared to 11.5% using PCR methods [13]. PCR is generally considered as an efficient tool to estimate the prevalence of AAT in affected areas [13], [14]. Thereby infections can be detected with more sensitivity and valuable information for prevalence studies provided [14]–[17]. The internal transcribed spacer (ITS) region of ribosomal DNA (rDNA) is a preferred target for universal testing because of its size variability among trypanosome species and subspecies. To discriminate the different trypanosome species in one step, primers (ITS1-CF and ITS1-BR) were designed targeting the ITS1 region. These show high diagnostic sensitivities and are capable of detecting all pathogenic trypanosomes in a single PCR with amplicon lengths below 720 bp. This is particularly helpful since approaches to combine conventional PCR reactions into a multiplex PCR for species differentiation are disappointing due to a decrease in sensitivity and non-specific PCR products (Njiru et al., 2005).

As prevalence studies of AAT are confronted with low levels of parasitaemia in chronically infected or trypanotolerant cattle, sensitivity is a major objective in test design and evaluation. Low levels of parasitaemia can only be detected with high-performance molecular diagnostic tests.

In trypanosomosis real-time PCR (qPCR) methods were described for *T. evansi* [18], [19], *T. brucei* [20] and *T. cruzi* [21], [22]. However, no qPCR for the simultaneous detection of the three species of AAT has been developed so far. In this study a new qPCR assay was designed and its performance compared to the conventional ITS1-PCR. In addition, information on morphological traits, biology and genetic background of the hosts was collected to identify risk factors for trypanosome infection.

The present study was conducted to (i) determine the prevalence and incidence of AAT in tsetse challenged areas of Burkina Faso, (ii) compare conventional and qPCR detection systems, (iii) determine levels of parasitaemia and (iv) evaluate the genetic background and host biology as risk factors.

## Materials and Methods

### Ethics statement

All blood samples from cattle were taken during routine veterinary examination by veterinarians or trained personnel. The participating owners of cattle provided their consent and agreed to fill out the survey form.

### Sample collection and DNA extraction

**Figure 1:**
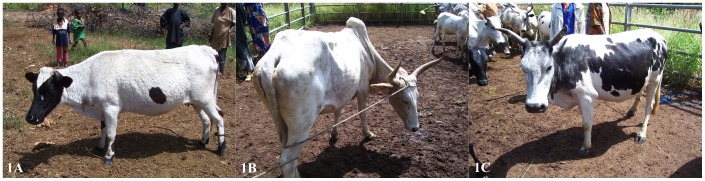
The three cattle types Baoulé, Zebu and Hybrids used in the study. Figure 1A. Trypanotolerant Baoulé breed (*Bos taurus*). Note the small horn size. Figure 1B. Trypanosusceptible Zebu type (*Bos indicus*). Note the large horn size and hump. Figure 1C. Hybrid cattle (Baoulé and Zebu crossbreds).

**Figure 2:**
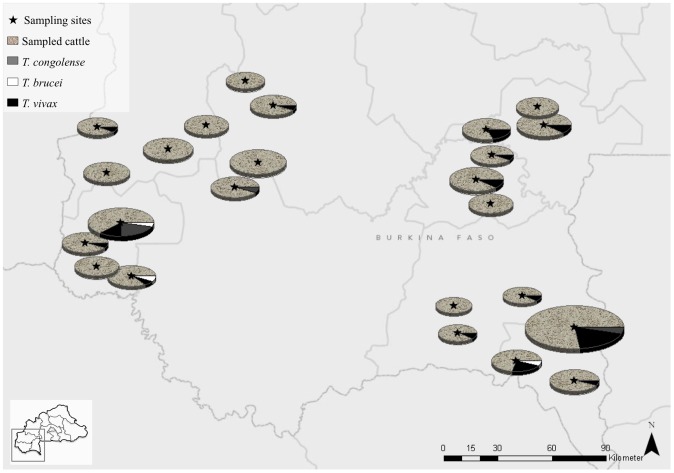
Map of the 24 sampled villages (12 villages in the West and 12 villages in the South-West) within the tsetse challenged areas of Burkina Faso. The size of the pie charts corresponds to the number of sampled and infected animals per site.

In this study 368 individuals from three cattle types, namely the indigenous Baoulé (n = 134; Figure 1A), the Zebu (n = 46; Figure 1B) and their hybrids (n = 188; Figure 1C) were sampled covering the three provinces Cascades, Hauts-Bassins and Sud-Ouest inside the tsetse belt of Burkina Faso. 12 villages from the West and 12 villages from the South-West of Burkina Faso were selected and cattle from six farms per village adding up to 72 different farms sampled (Figure 2). Purebred Zebus are rarely held in tsetse challenged areas due to their greater risk of severe disease. Therefore the sample set was not equally distributed within the three populations but reflects the actual population structure in southern Burkina Faso. In addition to the 368 animals, 28 Zebu cattle from the North (Seguenega n = 8, Marmisga n = 8, Yako n = 7, Nommon n = 5) were used to screen for mechanical trypanosome transmission. Breed assignment was performed based on farmer's information, asking also for the breed status of the two parents and records confirmed by body measurements (height at withers and chest circumference). In addition information on the genetic background was made available [3].

Whole blood was collected, 500 µL spotted onto each Whatman-FTA card sample area (GE Healthcare, Wisconsin, USA) air dried and stored at room temperature for subsequent DNA extraction. After transfer to the Vetmeduni Vienna, three discs of 3 mm each were taken from spotted blood on a total of 368×3 FTA cards and used for DNA extraction according to Whatman FTA Protocol BD08 (www.whatman.com). For improved DNA yield a Whatman FTA Purification Reagent containing 60 µg/ml Proteinase K was added to the disks and incubated overnight. On the next day washing steps with Whatman FTA Purification Reagent without Proteinase K, 0.3 M sodium carbonite, 0.5% SDS and TE^−1^ buffer were performed. Ultimately, the disks were placed into a new tube, incubated at 95°C for 15 min in 120 µl 1% PCR elution buffer and the supernatant collected. In addition to blood samples, we furthermore collected data on several variables with potential importance for the acquisition of trypanosome infections. Positive controls for *T. congolense Savannah*, *T. brucei brucei* and *T. vivax* were generously provided by the International Atomic Energy Agency (IAEA) and the concentration was spectrophotometrically determined using Ultrospec 2000 (GE Healthcare, Wisconsin, USA).

### Conventional PCR

**Figure 3:**
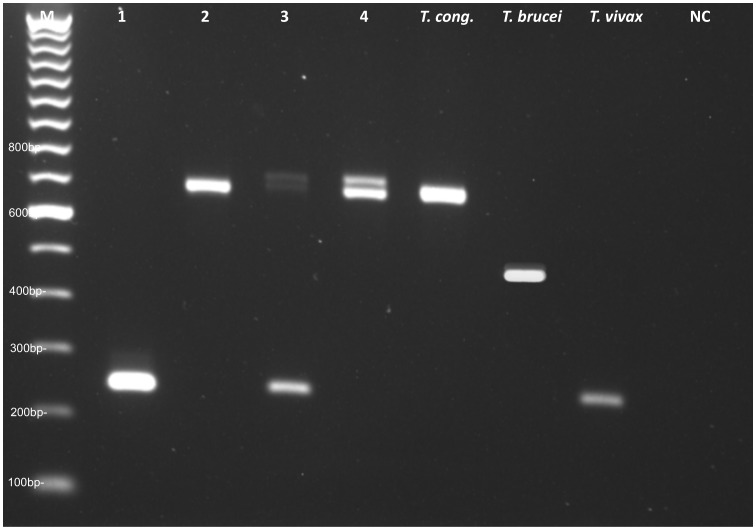
Gel electrophoresis of infected animals and reference DNA samples amplified with ITS1-CF and ITS1-BR. Lane 1: *T. vivax* (250 bp), lane 2: *T. congolense* – unspecified subtype (700 bp), lane 3: *T. congolense Forest* and *Savannah* (700 and 710 bp) and *T. vivax* (250 bp), lane 4: *T. congolense Forest* and *Savannah* (700 and 710 bp), NC (negative control).

The following primers were used to amplify the trypanosome ITS1 gene: ITS1 CF 5′-CCG GAA GTT CAC CGA TAT TG-3′ and ITS1 BR 5′-TTG CTG CGT TCT TCA ACG AA-3′ [16] resulting in species specific size products (Figure 3). PCR was performed in a 25 µL volume containing 5 µL genomic DNA, 600 nM of each primer, 5× PCR buffer (including 3 mM MgCl_2_), 0.8 mM dNTPs, and 1 U of Taq DNA polymerase (Go Taq, Promega, Madison, USA). PCR cycle conditions consisted of an initial denaturation step at 94°C for 3 min, 30 cycles of 94°C for 30 s, 55°C for 30 s, and 72°C for 30 s, followed by a final extension step at 72°C for 10 min. The amplified products were detected by electrophoresis on a 2% agarose gel (peqGOLD, PeqLab, Erlangen, Germany) stained with ethidium bromide.

### Sequencing

Species specific PCR was applied for the sequencing of selected positive samples for verification purposes. TCF1 and TCF2 for T. *congolense Riverine/Forest* and TCS1 and TCS2 for *T. congolense Savannah* [17] were used. PCR products were purified using Illustra ExStar 1-Step (GE Healthcare, Wisconsin, USA). Two samples with weak bands on the agarose gel were cloned into a TOPO vector (LifeTech Austria, Vienna, Austria) and sequenced. The resulting sequences were BLASTed using the basic BLAST search tool and aligned using CodonCode Aligner (version 2.0.6).

### qPCR

In the qPCR assay, new primers and probes for the simultaneous detection of all three AAT species were designed (Table 1). Trypanosome ITS1 sequences were selected from NCBI database (www.ncbi.nlm.nih.gov), including *T. congolense* (GI:1040856, GI:1040860 - *Forest*-type, GI:1040858 - *Kilifi* type), *T. brucei* (GI:14276830–14276836) and *T. vivax* (GI:1040857). The sequences were aligned in CodonCode Aligner and primers and TaqMan probes designed with Primer Express software version 2.0. The probes were placed spanning species specific sequences of the ITS1 region for subsequent species discrimination. The universal primers and species specific fluorescent labelled probes allowed the detection of parasite DNA upon binding on the ITS1 gene. The specificity of the probes and primers was evaluated using BLAST searches and Primer BLAST respectively. A simultaneous amplification of the cattle specific toll-like-receptor-8 (TLR-8) gene was run as inhibition control to detected false negative results (Table 1). qPCR was performed in a 25-µL volume containing 5 µL genomic DNA, 200 nM of each primer, 160 nM of each trypanosome probe, 120 nM of the TLR-8 probe, 5× PCR buffer, 6 mM MgCl_2_ 0.8 mM dNTPs, and 1 U of Taq DNA polymerase (GoTaq Hot Start Polymerase, Promega, Madison, USA). All three trypanosome FAM-labelled probes were added in equal amounts. The qPCR cycle protocol started with the initial denaturation at 95°C for 10 min, 45 cycles of 95°C for 30 s, 61°C for 1 min, and ended with 72°C for 1 min. qPCR was performed in a Strategene Mx3000P (Agilent, Santa Clara, USA) thermal cycler.

**Table 1:** Primers and probes of the novel AAT qPCR and internal control.

Name	Primer/Probe sequence	Size (nt)	Product size	Sequence identity
Tryps_KS-for	5′-CGT GTC GCG ATG GAT GAC TT-3′	20	120 bp	NA
Tryps_KS-rev	5′-CAA ACG GCG CAT GGG AG-3′	17	120 bp	NA
Tryps_KS-T.cong-p	FAM 5′-TTG CAG AAT CAT CAC ATT GCC CAA TCT TTG-3′ BHQ1	30		T. evansi (EF546057) 97%, T. brucei (FJ712717) 96%
Tryps_KS-T.brucei-p	FAM 5′-TGC GAT AAG TGG TAT CAA TTG CAG AAT CAT TTC A-3′ BHQ1	34		T. evansi (JN896755) 100%
Tryps_KS-T.vivax-p	FAM 5′- ATG ACC TGC AGA ACC ACT CGA TTA CCC AGT-3′ BHQ1	30		NA
TLR8-for	5′-TGTTTAGAGGAAAGGGATTGGG-3′	22	69 bp	NA
TLR8-rev	5′-TTGGTTGATGCTCTGCATGAG-3′	21	69 bp	NA
TLR8-p	HEX 5′-CCCGGGTCTAGCCATCATCGACAA-3′ BHQ1	24		NA

All amplifications were reproduced in duplicates whereas triplicates were used for the standard curve and limit of detection. Quantification cycle (Cq) values of ≤38 were regarded as potentially positive and those samples used for further analyses. For species differentiation all potentially positive samples were applied to single-plex qPCR assays containing only one species specific probe. Alongside, the level of parasitaemia was identified in the single-plex assays. Using serial dilutions of trypanosome DNA the sensitivity in detecting and quantifying trypanosome infections was analysed. For the limit of detection serial 10-fold dilutions ranging from 20 ng to 0.2 fg were tested for each trypanosome positive control. This covers 10^5^ to 0.001 parasite equivalents when considering that one parasite cell consists of approximately 200 fg of DNA [21]. The assay performance was tested individually for each probe and efficiency, slope and RSq values checked over a standard calibration curve covering a fivefold 4× dilution series. The values were calculated using the MxPro - MX3000P v 4.01 software. The specificity of the assay was tested with animals positive for *Babesia divergens* (n = 2) and *Theileria* sp. (n = 2).

### Statistics

We identified variables potentially important for the acquisition of trypanosome infections and investigated associations of these with infection events. 72 farmers were interviewed with questionnaires and morphometric measurements of the cattle taken.

A Chi squared test was performed in SAS version 9.2. [23] to test for associations between cattle breed and AAT infection.

In addition a logistic regression model was calculated using breed, coat colour, gender, age, trypanocidal treatment history and type of trypanosome prevention as fixed effects and village as random effect (Table 2).

**Table 2:** Positive tested animals and corresponding biological and husbandry parameters of cattle.

ID	location	village	breed	coat colour	gender	age	previous treatment	prevention	Infection	parasite equivalents
BD224	South-West	Djonkargo	ZB	bw	M	4 m	-	-	TV	0.065
BD22	South-West	Dollo	B	brw	M	1 y	✓ (Veriben)	-	TV	35.25
BD41	South-West	Dollo	B	b	M	2 y	✓ (Trypamidium, Diminazen)	-	TV	0.38
HN41	West	Nasso	ZB	w	F	15 y	✓ (Trypamidium, Diminazen)	-	TV	0.07
HT312	West	Toussiana	ZB	bw	M	3 y	✓ (Trypamidium, Berenil)	-	TCS	NA
IB34	South-West	Bouni	ZB	brw	M	1 m	-	-	TV	0.005
IB38	South-West	Bouni	B	br	M	1 y	✓ (Berenil)	-	TV	NA
IB42	South-West	Bouni	B	bw	M	1 y	✓ (Trypamidium, Berenil)	✓ (fly control)	TV	0.125
ID32	South-West	Dano	B	bw	M	1.5 y	✓ (Trypamidium, Berenil)	-	TV	0.145
ID34	South-West	Dano	B	bw	M	10 m	✓ (Trypamidium, Berenil)	-	TV	2.275
KK52	West	Koloko	ZB	wb	M	2 y	-	-	TV	0.090
LK27	West	Kasseguera	ZB	w	M	1 y	✓ (Trypamidium)	-	TB	NA
LK52	West	Kasseguera	ZB	w	M	1 m	-	-	TV	0.080
LL52	West	Loumana	ZB	wb	M	2 y	✓ (Trypamidium, Berenil)	-	TV	2.341
LS14	West	Sindou	ZB	wb	M	1.5 y	✓ (Securidium)	-	TCS	0.005
LS25	West	Sindou	ZB	w	F	8 y	✓ (Survidim)	-	TC	0.420
									TB	0.840
LS26	West	Sindou	ZB	wb	M	2 y	✓ (Survidim)	-	TC	0.185
LS38	West	Sindou	ZB	wb	M	1 y	✓ (Veriben)	-	TC	0.050
									TV	0.265
LS43	West	Sindou	ZB	w	F	5 y	✓ (Trypamidium, Isometamidium)	-	TCS	0.010
LS44	West	Sindou	ZB	w	M	2 y	✓ (Trypamidium, Isometamidium)	-	TV	0.005
LS46	West	Sindou	ZB	wbr	M	10 m	✓ (Trypamidium, Isometamidium)	-	TV	NA
LS52	West	Sindou	ZB	brw	M	1 y	✓ (Trypamidium, Isometamidium)	-	TCS	0.025
LS58	West	Sindou	ZB	wbr	M	6 m	✓ (Trypamidium, Isometamidium)	-	TV	2.735
NB41	South-West	Batie	B	b	M	6 m	-	-	TV	NA
NL17	South-West	Kour	Z	w	M	2 m	-	-	TCF	0.015
									TV	0.805
NL29	South-West	Kour	Z	wbr	M	4 m	-	-	TV	0,66
NL311	South-West	Kour	Z	w	M	10 m	✓ (Trypamidium, Veriben)	-	TV	10.150
NL31	South-West	Kour	Z	wb	M	6 m	✓ (Trypamidium, Veriben)	-	TV	NA
NL312	South-West	Kour	Z	w	F	9 y	✓ (Trypamidium, Veriben)	-	TCS	0.065
									TV	NA
NL39	South-West	Kour	Z	w	M	1.5 y	✓ (Trypamidium, Veriben)	-	TV	0.005
NL411	South-West	Kour	Z	brw	M	3 m	-	-	TV	0.005
NL61	South-West	Kour	Z	w	M	4 m	-	-	TV	0.035
NL64	South-West	Kour	Z	brw	M	11 y	-	-	TV	NA
NL65	South-West	Kour	Z	w	F	3 m	-	-	TV	NA
NL69	South-West	Kour	ZB	w	M	7 m	✓ (Trypamidium, Veriben)	-	TV	0.805
NM15	South-West	Midebdo	B	b	M	10 m	✓ (Trypamidium, Berenil)	-	TV	0.085
NM19	South-West	Midebdo	B	bw	M	1.5 y	✓ (Trypamidium, Berenil)	-	TV	0.065
NM22	South-West	Midebdo	B	bw	M	3 m	-	-	TV	3.845
NM26	South-West	Midebdo	B	b	M	0.5 m	-	-	TB	NA
									TV	0.035
PG35	South-West	Gaoua	B	bw	M	2 y	-	-	TV	0.180
PT24	South-West	Takouloula	B	b	M	4 m	-	-	TV	21.650

Breed is Baoulé (B), Zebu (Z) or hybrids (ZB). Coat colour is black (b), brown (br), brown-white (brw), black-white (bw), white (w), white-black (wb), white-brown (wbr). Gender is male (M) or female (F). Age is indicated in years (y) or months (m). Trypanosome infections are *T. congolense* – unspecified subtype (TC), *T. congolense Savannah* (TCS), T. *congolense Forest* (TCF), *T. brucei* (TB) and *T. vivax* (TV). Parasite equivalents calculated according to (Duffy et al., 2009) considering that one parasite cell consists of approximately 200 fg of DNA.

## Results

### PCR results

The analysis of the 368 field samples (134 Baoulé, 46 Zebu, 188 Baoulé×Zebu hybrids) with conventional PCR revealed an overall trypanosome prevalence of 10.87% (40/368). 40 animals were positive for trypanosome DNA. Of these animals two showed multiple infections. Altogether 42 infections in 40 different animals could be detected.

*T. congolense* (including *Savannah* and *Forest* subspecies) gave an approximate band size of 700 bp (Figure 3) and were detected in nine cases. *T. vivax* (250 bp) was detected in 33 cases. No infection with *T. brucei* (480 bp) was discovered with conventional PCR. The highest infection incidences were identified in Zebu cattle with 10 of 46 Zebus (21.74%) infected, one of which had multiple infections (*T. congolense*/*T. vivax*). None of the 20 Baoulé from the West was infected, in contrast to 13 Baoulé (11.40%) from the Southwest adding up to a total prevalence in Baoulé of 9.70% (13/134). Of the 188 hybrids, 17 animals (9.04%) were infected. As expected, no animal from the North regions outside the tsetse belt was positive.

### qPCR results

By analysing serial dilutions no signal could be obtained from samples containing ≤2 fg DNA. This detection limit corresponds to 0.01 parasite genomic equivalents. To increase the specificity of the *T. brucei* assay and avoid cross-reactions with the *T. congolense* probe, the primer annealing temperature was increased from 61°C to 65°C. The assay performance of the three probes was assessed in separate reactions. The *T. congolense* assay efficiency was 97.0%, slope Y = −3.395 and RSq: 0.995, the *T. brucei* assay efficiency was 97.5%, slope Y = −3.384 and RSq: 0.988 and the *T. vivax* assay efficiency was 98.3%, slope Y = −3.364 and RSq: 0.986. To test for specificity of the qPCR assay *Babesia divergens* and *Theileria* sp. positive samples were analysed but no cross-reactivity with related protozoan blood parasites could be detected. After establishing the three assays, the probes were multi-plexed and a total of 368 blood samples from three populations analysed in duplicates. With qPCR a total of 41 animals were tested positive for trypanosome DNA. Of these animals five showed multiple infections. Altogether 46 infections in 41 different animals could be detected (Table 2). Our results indicate that the qPCR results are comparable to the conventional PCR results with a concordance of 91.30%.

**Figure 4:**
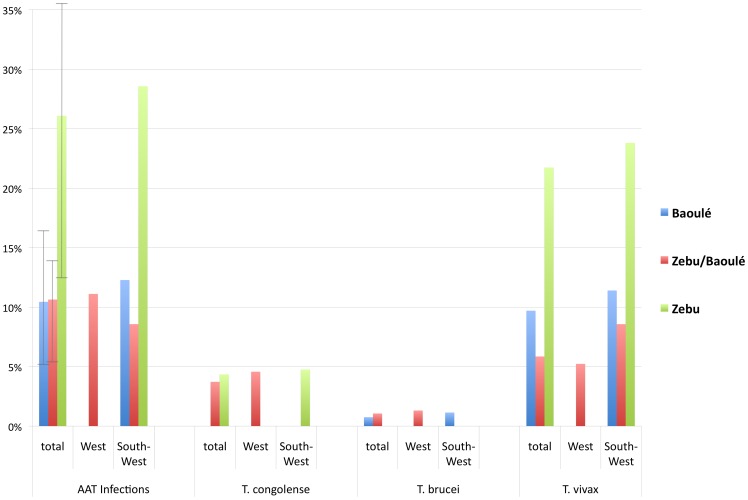
Prevalence of African Animal Trypanosomosis (AAT) according to Burkina Fasa region, trypanosome species and cattle type.

All positive samples from conventional PCR were verified with qPCR. The overall rate of prevalence detected with qPCR increased to 11.14% (41/368) with 21.74% (10/46) of Zebus infected. Baoulé and hybrids show an infection rate of 9.70% (13/134) and 9.57% (18/188), respectively (Figure 4).

When analysing the parasitic load of the 46 detected infections, eleven (two *T. congolense*, two *T. brucei* and seven *T. vivax*) specimens were PCR-positive but outside the linear range of the standard curve, indicating a low level of parasitaemia (Table 2). A total of 39 sequences were obtained by sequencing the qPCR products directly or after applying species specific primers to verify the positive results. Of these 14 fulfilled the submission criteria (≥200 bp) and were deposited in Genbank (JX910370–JX910383). In case of multiple bands (*T. congolense* subspecies), only the species verified by sequencing was regarded as positive. In our study *T. vivax* is the dominant species across all analysed area with 34 animals infected (9.24%) as compared to *T. congolense* (nine animals; 2.44%) and *T. brucei* (three animals; 0.82%; Figure 2 and 4). None of the animals from the North were positive.

### Statistics

For the statistical analysis the variables village, breed, coat colour, gender, age, trypanocidal treatment history and type of trypanosome prevention were used (Table 2).

The results of the questionnaire asking for the trypanocidal treatment history of the cattle revealed that isometamidium chlorides (Trypamidium, Securidium) and diminazene aceturates (Berenil, Diminaveto, Veriben, Trypadim, Survidim) were applied either by the farmers or by skilled personnel. The types of trypanosome prevention were fighting flies or traditional methods (e.g. scarification, traditional medicines).

A chi-squared test linking breeds with infection events (rates of 21.74% for Zebu, 9.70% for Baoulé and 9.57% for hybrids, respectively) gave a near-significant result (p = 0.0507). When comparing Zebu with Baoulè and Zebu with hybrids separately, the results were significant (Zebu – Baoulé *P* = 0.0349 and Zebu – hybrids *P* = 0.0227). However, after correction for multiple testing the results were not significant. In the logistic regression model no significant effects of the seven analysed variables potentially important for the acquisition of trypanosome infections could be detected at a significance level of *P*<0.05.

## Discussion

AAT is a major constraint on livestock production in Africa [2]. To assess the actual impact of the disease, AAT prevalence was associated with potential risk factors of biology and husbandry of cattle [24], [25]. Such studies rely on accurate detection methods for circulating parasites. qPCR strategies offer a high level of analytical sensitivity and can be multiplexed with several fluorescent labelled probes. Therefore qPCR is regarded as a key laboratory tool for monitoring parasitic infections [21].

This study introduces a novel qPCR assay for the simultaneous detection of the three AAT species *T. congolense*, *T. brucei* and *T. vivax*. The ITS1 gene, present in around 200 copies per genome, has already been used in conventional PCR studies to detect trypanosome DNA at a dilution equivalent to less than one parasite per ml blood [15]. By using this region we created a highly sensitive, reproducible and specific qPCR assay for the detection and quantification of AAT parasites in cattle blood. The overall prevalence of all 368 analysed animals from 72 farms in Burkina Faso, detected with the novel qPCR assay was 11.14%. Previously published studies have reported a similar prevalence of 7.54% [26] and 11.5% [13] in South-Western areas of Burkina Faso.

As shown in our study, the results of the novel qPCR are comparable to the conventional PCR with a concordance of 91.30%. However, three additional co-infections and one additional infected animal were detected in the qPCR compared to conventional PCR results (Table 3). The discrepant results between PCR and qPCR, reflected by the slightly better assay performance of the qPCR, can be explained by low levels of parasitaemia, probably below the detection threshold of conventional PCR. We see that the samples with a positive qPCR result but negative for the conventional PCR present late Cq values and quantities outside the linear range. Positive qPCR results with low levels of parasitaemia have been previously observed where quantification of the parasitic load was unsuccessful due to values outside the linear range of the standard curve [18]. One limiting factor in parasite detection could be the FTA-card sample storage system. FTA-cards offer a convenient method for large-scale prevalence studies [15]. However the parasite DNA is not evenly spread across the filter-paper resulting in inaccurate results [27]. Thus, not only the diagnostic method, but also the procedure of sample preparation is a driving factor for improved sensitivity in epidemiological screening surveys. In our study we used three discs of three FTA card sample areas for DNA extraction. The median level of parasitic load was highest in Baoulé (0.135 parasite genomic equivalents/rxn), followed by Zebus (0.010 parasite genomic equivalents/rxn) and hybrids (0.068 parasite genomic equivalents/rxn), calculated according to Duffy et al., 2009. The high parasitic load in Baoulé can be accounted to two highly infected animals BD22 and PT24, probably due to acute infections. It is known that taurine breeds are more tolerant to trypanosome infections and can better cope with the anaemia following high parasitaemia levels [4], [11], [28], [29]. The parasitic load was highest in Baoulé and in hybrid cattle with similar rates of infection (9.70 vs. 9.57%). When considering that all included animals were described as healthy by their owners, these results suggest that crossbreeding in the analysed tsetse challenged regions of Burkina Faso was successfully applied as a management tool of AAT. Zebus are more susceptible and twice as often infected (21.74%) compared to Baoulé and hybrids. They show significantly higher infection rates compared to Baoulé (*P* = 0.0349). Trypanotolerance is a multilocus trait with a complex hereditary mode, particularly under field conditions and investigations using quantitative trail loci (QTLs) are still on-going [30]–[33]. In a complex statistical model linking breed, village, coat colour, gender, age, trypanocidal treatment history and type of trypanosome prevention, no risk factor could be associated with the infection status in our study. However, for reliable calculations of complex models a higher sample size and higher numbers of infected animals would be needed. The most prevalent AAT species in our sample set was *T. vivax* with 9.24% compared to *T. congolense* (2.44%) and *T. brucei* (0.82%; Figure 2 and 4). This is consistent with recent results from the Comoe district in Burkina Faso which show that trypanosome infections were predominantly due to *T. vivax* [26], [34]. Interestingly, in Sideradougou *T. congolense Savannah* type was the predominant species [13]. When looking at Toussiana village located approximately 44 km (27 miles) from Sideradougou in our dataset, we also detected *T. congolense Savannah* type (animal HT312) without any other trypanosome species present. It is known that different *Glossinidae* have diverse vector competences for different trypanosome species [35], [36]. *G. morsitans submorsitans* is regarded as a good vector for *T. congolense Savannah* [37], [38]. An in-depth vector and host surveillance program in this area would be helpful to shed more light on tsetse type abundance and *T. congolense Savannah* infections.

**Table 3:** Comparison matrix of PCR and qPCR results.

qPCR detection	no. of detected infections with PCR					total no. of samples
	neg	*TC*	*TB*	*TV*	*TCV*	
neg	327					327
*TC*		5				5
*TB*	1					1
*TV*				30		30
*TCB*		1				1
*TCV*		1			2	3
*TBV*				1		1
total	328	7		31	2	368

neg.: negative tested samples, *T. congolense* – unspecified subtype (TC), *T. brucei* (TB), *T. vivax* (TV), *T. congolense* and *T. brucei* (TCB) multiple infections, *T. congolense* and *T. vivax* (TCV) multiple infections, *T. brucei* and *T. vivax* (TBV) multiple infections.

When looking further at the distribution of AAT incidences we noticed one interesting region in Western Burkina Faso. The village of Sindou was sampled with 14 cattle, all belonging to the hybrid group. This village has the highest infection rate in the study, with nine out of 14 cattle being infected. We thus assume that the infection pressure is exceptionally high in this area. In fact, Sindou located in the Cascades province, was affected by increased AAT incidences in the year 2006 resulting in losses of many heads of cattle (Soudre pers. comm.).

The present assay can be placed in line with established detection systems for various trypanosome species like *T. brucei* [20], [39], *T. cruzi* [40], [41] and *T. evansi* [18], [19]. Our novel qPCR assay can detect the three AAT species *T. congolense*, *T. brucei* and *T. vivax* simultaneously. The separate probes can be labelled with different dyes and species determination achieved in only one multiplex qPCR reaction. This method could even be applicable to distinguish trypanosome species not only in cattle hosts, but also in tsetse flies, where morphological traits of the parasite and tissue localisations in the vector are not accurate enough to provide reliable diagnosis [42], [43]. The taxonomic groups *T. congolense Tsavo* and *Kilifi* type and *T. simiae* have never been identified in vectors and hosts of Burkina Faso [7], [37]. These species show distinct sequence variations and are not amplified by our novel qPCR assay.

In summary, the novel qPCR technique for the simultaneous detection of the three AAT species *T. congolense*, *T. brucei* and *T. vivax* offers a simple, fast and sensitive method compared to conventional PCR. Contaminations can be minimized since single-round PCR reactions are performed and false negative results detected with internal controls. Permanent carrier animals with low parasitaemia levels can be detected and parasitic loads quantified. Detecting the level of parasitaemia is relevant in epidemiological studies to investigate host-vector-pathogen interactions.

Our results suggest that breed is a risk factor for AAT infection in contrast to village, coat colour, gender, age, trypanocidal treatment history and type of trypanosome prevention, where no correlations could be found. Zebu cattle are more likely to be infected compared to Baoulé and hybrids. Thus, indigenous taurine cattle breeds, like the Baoulé, provide a unique and valuable genetic resource which needs to be preserved.

## Supporting Information

Figure S1 Standard curves of the *T. congolense, T. brucei* and *T. vivax* qPCR assay.
(TIF)

Figure S2 Study design for the detection of African Animal Trypanosomosis in Burkina Faso.
(TIF)

Figure S3 STARD checklist for diagnostic tests.
(DOC)
