# Correction: A Novel qPCR Assay for the Detection of African Animal Trypanosomosis in Trypanotolerant and Trypanosusceptible Cattle Breeds

**DOI:** 10.1371/journal.pntd.0003461

**Published:** 2014-12-19

**Authors:** The PLOS Neglected Tropical Diseases Staff

In Table 1, the Primer/Probe sequence for Tryps_KS-rev is incorrect. The correct sequence is: CTCCCATGCGCCGTTTG.
