# Supplementary figures and images for: A Novel qPCR Assay for the Detection of African Animal Trypanosomosis in Trypanotolerant and Trypanosusceptible Cattle Breeds
Source: PLoS Negl Trop Dis. 2013 Aug 15;7(8):e2345. doi: 10.1371/journal.pntd.0002345 (PMC3744421; doi:10.1371/journal.pntd.0002345)

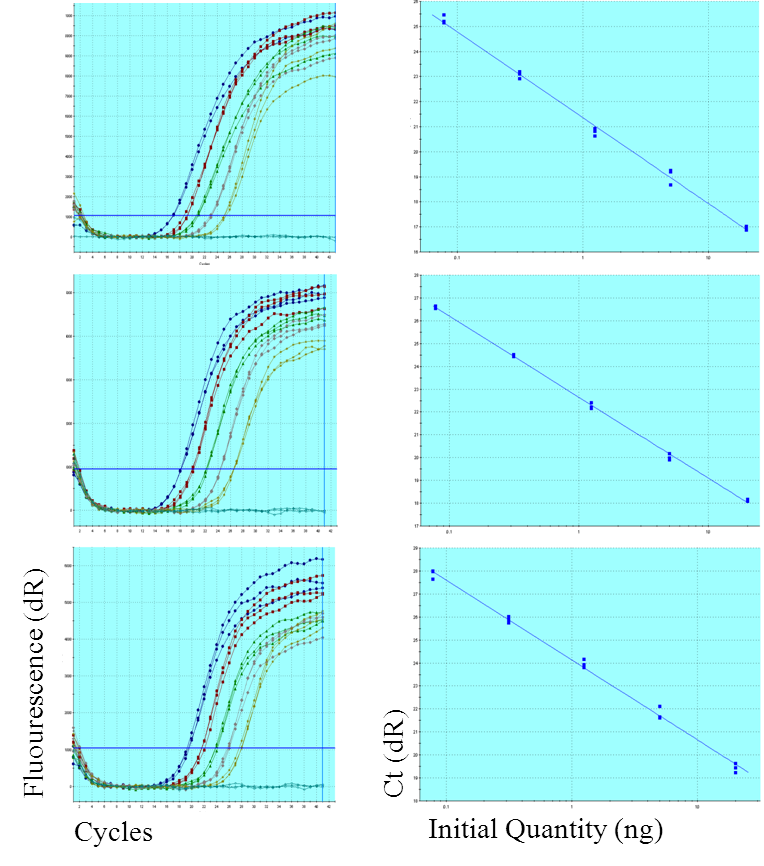

Supplement: Figure S1 — Standard curves of the T. congolense, T. brucei and T. vivax qPCR assay. (TIF) [file pntd.0002345.s001.tif]

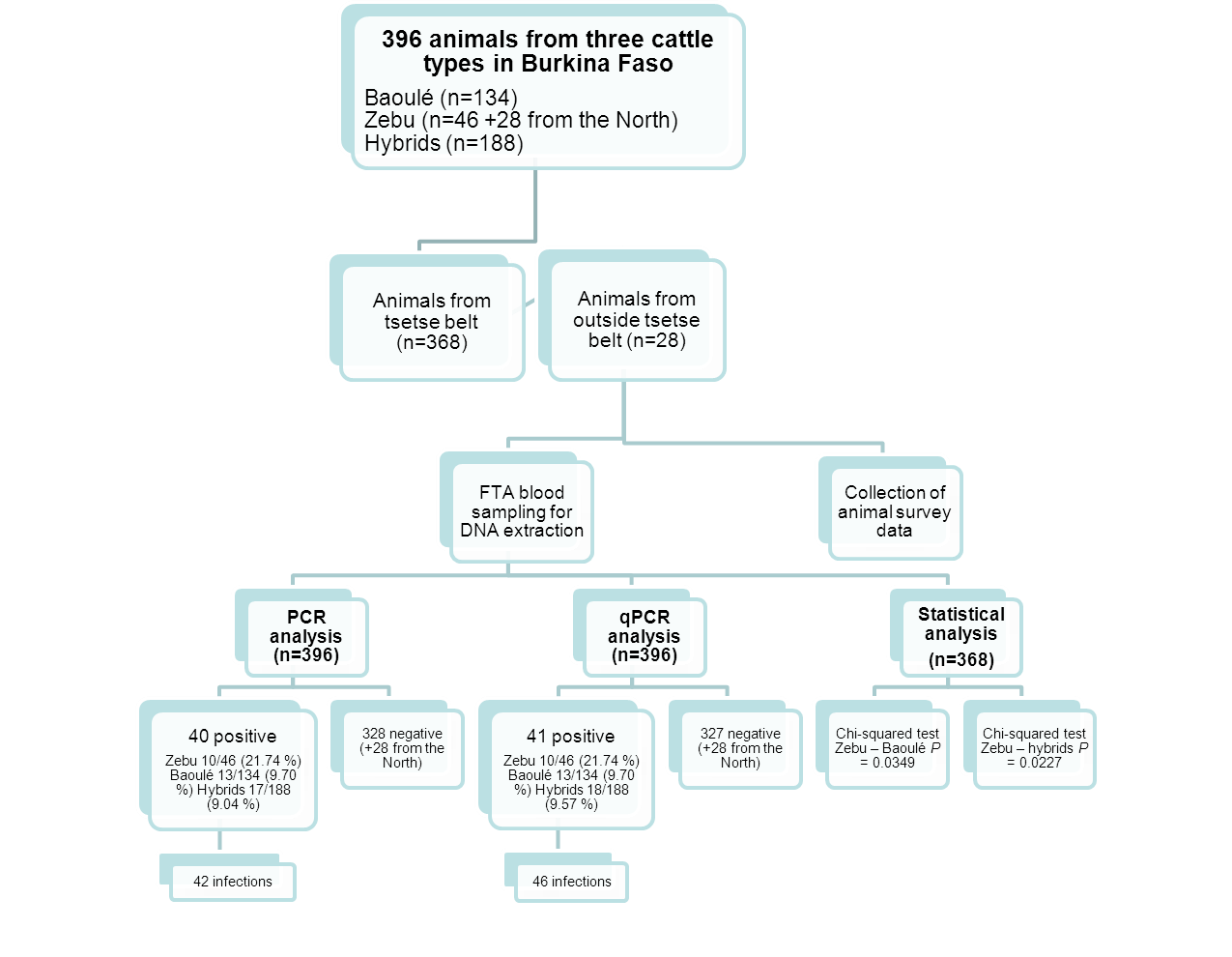

Supplement: Figure S2 — Study design for the detection of African Animal Trypanosomosis in Burkina Faso. (TIF) [file pntd.0002345.s002.tif]
